# UHMK1 aids colorectal cancer cell proliferation and chemoresistance through augmenting IL-6/STAT3 signaling

**DOI:** 10.1038/s41419-022-04877-8

**Published:** 2022-05-02

**Authors:** Xuedi Gao, Wenfang Bao, Jin Bai, Kailing Fan, Li Li, Yandong Li

**Affiliations:** Department of Oncology, Shanghai East Hospital, Tongji University School of Medicine, Shanghai, 200120 China

**Keywords:** Oncogenes, Cell signalling, Cell growth

## Abstract

UHMK1, a serine/threonine kinase with a U2AF homology motif, is implicated in RNA processing and protein phosphorylation. Increasing evidence has indicated its involvement in tumorigenesis. However, it remains to be elucidated whether UHMK1 plays a role in the development of colorectal cancer (CRC). Here, we demonstrated that UHMK1 was frequently upregulated in CRC samples compared with adjacent normal tissue and high expression of UHMK1 predicted poor outcomes. Knockdown of UHMK1 by siRNAs restrained CRC cell proliferation and increased oxaliplatin sensitivity, whereas overexpression of UHMK1 promoted CRC cell growth and oxaliplatin resistance, suggesting that UHMK1 plays important oncogenic roles in CRC. Mechanistically, we showed that UHMK1 had a significant effect on IL6/STAT3 signaling by interacting with STAT3. The interaction of UHMK1 with STAT3 enhanced STAT3 activity in regulating gene transcription. Furthermore, we found that STAT3 could in turn transcriptionally activate UHMK1 expression in CRC cells. The complementary experiments for cell growth and oxaliplatin resistance indicated the interdependent relationship between UHMK1 and STAT3. Thus, these collective findings uncovered a new UHMK1/STAT3 positive feedback regulatory loop contributing to CRC development and chemoresistance.

## Introduction

Colorectal cancer (CRC) has been indicated as the second deadliest malignancies, and it is also one of the most commonly diagnosed malignancies of the digestive system. The incidence of CRC is on rise worldwide, emphasizing the urgency and importance of deeply exploring its initiation and progression mechanisms [1, 2]. There are many factors involved in the pathogenesis of CRC, among which, chronic intestinal inflammation is one of the most important ones [3]. Multiple proinflammatory signaling pathways have been implicated in the transformation of chronic intestinal inflammation to CRC, including the NF-κB, IL-23/Th17, COX-2/PGE2, and IL-6/STAT3 pathways [4–8]. The dysregulation of any factor in these signaling pathways may lead to aberrant biological behaviors in colorectal tissue, such as tumor cell transformation, accelerated cell proliferation, growth, metastasis, and enhanced drug resistance and immune escape [9–11]. The question of how these factors and signaling pathways are activated and maintained in CRC is not well understood yet.

U2AF homology motif kinase 1 (UHMK1) is a unique serine/threonine kinase (Ser/Thr kinase) that possessed a C terminal domain with a U2AF homology motif (UHM) [12]. This domain is an RNA recognition motif (RRM) domain homologous to protein binding RRMs found in U2AF65 and U2AF35 [13]. The two proteins act as the large submit (U2AF65) and the small submit (U2AF35) of the U2 auxiliary factor (U2AF) heterodimer [14]. Other members in UHM families are also nuclear proteins, such as PUF60, HCC1, and SPF45 [15]. The UHM family members are known to participate in constitutive or alternative pre-mRNA splicing and consequent other multiple biological processes [16, 17]. With the U2AF homology motif domain, UHMK1 phosphorylates the splicing factor SF1 on the SPSP motif, suggesting an important role in spliceosome assembly and RNA splicing [18]. Besides, UHMK1 can positively regulate cell cycle progression with its kinase domain. Following mitogen stimulation, UHMK1 increases the phosphorylation level of p27 at serine 10, subsequently promoting cell cycle re-entry in vascular smooth cells [19, 20].

The abnormally elevated UHMK1 has been reported to be a potential oncogenic factor in different types of human cancers. Studies have done through ovarian cancer to gastric cancer [21–24]. However, the expression, function, and regulatory mechanism of UHMK1 in CRC remain unclear. Herein, in the present study, we described the expression of UHMK1 by online database mining and immunohistochemical staining in a CRC tissue microarray. We examined its role in cell proliferation and chemoresistance in vitro and in vivo. More importantly, we set up a link between UHMK1 and IL6/STAT3 signaling, proposing UHMK1 could augment this signaling pathway by a positive feedback loop to promote CRC development and chemoresistance.

## Materials and methods

### Human tissue microarray (TMA)

The colon cancer tissue microarray (#HColA180Su19) was purchased from Shanghai Outdo biotech Co. Ltd, China, which contains 86 paired colon cancer tissues and corresponding adjacent normal tissues and 8 cases of independent colon cancer tissues. Immunohistochemical staining (IHC) of UHMK1 (11624-1-AP, at a dilution of 1:200, Proteintech, Wuhan, China) was carried out according to the commercial protocol (Outdo Biotech). The immunostaining was independently evaluated by two pathologists. The statistical analysis was performed as described in our previous publication [25]. In addition, another two TMAs (#HColA030PG06, each contains 30 cases of colon cancer tissues) were obtained from Shanghai Outdo biotech for examining the expression correlation between UHMK1 and STAT3. STAT3 antibody (#9139, Cell Signaling Technology, USA) was used at a dilution of 1:500 for IHC. The research was approved by the Medical Ethics Committees of Shanghai East Hospital, Tongji University School of Medicine.

### Cell culture

The human CRC cell lines RKO, HCT116, Caco-2, HT29, LoVo, DLD-1, and SW480 were purchased from Shanghai Cell Bank of Chinese Academy of Sciences. Before use, all cell lines were authenticated using short tandem repeats (STRs) sequencing. The HEK293T cell was originally from ATCC and stocked in our laboratory. These cells were cultured in DMEM, RPMI-1640, Ham’s F-12K or McCoy’s 5 A medium supplemented with 10% fetal bovine serum, 100 U/mL penicillin and 100 mg/mL streptomycin in a humidified atmosphere containing 5% CO_2_ at 37 °C. The inhibitors of STAT3, SH-4-54 and BP-1-102 (#S7337 and #S7769) were purchased from Selleck, Shanghai, China.

### RNA isolation and qRT-PCR (quantitative real-time PCR)

Total RNA was extracted from CRC cells using TRizol regent (Sigma-Aldrich, Germany) according to the manufacturer’s protocol. RNA (2 μg) was reverse-transcribed using a PrimeScript RT reagent kit (TaKaRa, Japan). qRT-PCR was performed using TB Green^®^ Premix Ex Taq™ kit (Takara, Japan) in an ABI 7500 PCR system (Applied Biosystems, USA). Each reaction was performed in triplicate. The mRNA level of each sample was normalized to β-actin via 2^-ΔCt^ method. Primer sequences used in this study are listed in Supplementary Table 1.

### Western blotting and Co-immunoprecipitation

The whole cell lysates were extracted with RIPA buffer (Merck Millipore, USA) plus protease inhibitor cocktail (Sigma-Aldrich, Germany) and phosphatase inhibitor cocktail was added when phosphorylated proteins need to be detected. Nuclear and cytoplasmic proteins were extracted using NE-PERTM Nuclear and Cytoplasmic Extraction Reagents (REF78833), Thermo Scientific, USA. The proteins were separated by SDS-PAGE, transferred onto a nitrocellulose filter membrane and incubated with specific primary antibodies. The final bands were visualized with Odyssey Infrared imaging system (Li-COR Biosciences, USA) after corresponding secondary antibodies incubations. The primary antibodies are as follows: UHMK1 (#11624-1-AP), GAPDH (#60004-1-Ig), c-MYC (#10828-1-AP) and FLAG-like tag (#66008-3-Ig) were obtained from Proteintech, Wuhan, China. HA-tag (#3724), STAT3 (#9139), p-STAT3 (#9145) and Cyclin D1 (#2922) were obtained from Cell Signaling Technology, USA. For immunoprecipitation, lysis buffer without SDS (25 mmol/L Tris-HCl pH 7.4, 150 mmol/L NaCl, 1% NP-40 nonyl phenoxypolyethoxylethanol and protease inhibitor cocktail) was used for protein preparation. The procedure was performed as previously described [26].

### RNA interference and gene overexpression

The small interference RNAs (siRNAs) against UHMK1 and STAT3 were chemically synthesized by GenePharma, Shang, China. The sequences for UHMK1: siUHMK1-1, 5’- AGAAGGCAAUCAGGAUGUAdTdT-3’; siUHMK1-2, 5’-AAGCAGUUCUUGCCGCCAGGAdTdT-3’. The sequences for STAT3: siSTAT3-1, 5’-GGUACAUCAUGGGCUUUAUdTdT-3’; siSTAT3-2, 5’-AACAUCUGCCUAGAUCGGCUAdTdT-3’. The sequences for a negative control: siNC, 5’-UUCUCCGAACGUGUCACGUdTdT-3’, is not targeted any annotated human gene. The lentivirual particles for UHMK1 knockdown were packaged and obtained from GenePharma, Shang, based on the siUHMK1-1 sequence. The pcDNA3.1-UHMK1-3×FLAG and pcDNA3.1-HA-STAT3 were purchased from PPL (Public Protein/Plasmid Library), Nanjing, China, for the overexpression of UHMK1 and STAT3. For the construction of STAT3 protein defective in DNA binding, the pcDNA3.1-HA-STAT3 plasmid was mutated by site-directed mutagenesis for generating L358A, Y359A, L360A and V366A mutant STAT3 protein. For stably expressing UHMK1, DLD-1 and SW620 cells were transfected transiently with pcDNA3.1-3×FLAG and empty vector, then cells were selected by G418 (800–1000 μg/ml) and single colonies were isolated and verified by western blotting. Plasmids or siRNAs were transfected into cells by Lipofectamine 3000 (Invitrogen, USA) following the manufacturer’s instructions.

### Cell proliferation, colony formation, and apoptosis assays

Cell growth curve and viability were measured by CCK-8 kit (Dojindo Laboratories, Japan) as the manufacturer’s protocol. For EdU proliferation assays, cells were incubated with 50 mM EdU for 2 h at room temperature and stained with Apollo^®^ 567 obtained from RiboBio, Guangzhou, China. The CRC cells were observed and counted with an inverted fluorescence microscope. Colony formation assays were done in line with previously reported in 6-well plates [25], 1000 cells/well. For drug sensitivity assays, cells were treated with or without oxaliplatin at indicated dose for 48 h and then suffered to flow cytometry analysis using Annexin V-FITC/PI apoptosis detection kit (Dojindo Laboratories, Japan) by a flow cytometer (BD Bioscience). All the experiments above were independently repeated for three times.

### Luciferase reporter assay

A 2 kb length promoter of UHMK1 was cloned into pGL3-basic luciferase reporter vector, and the putative binding sites of STAT3 were mutated by site-directed mutagenesis. The wild-type and mutant constructs were respectively co-transfected into 293 T or CRC cells with Renilla luciferase plasmid pRL-SV40, siSTAT3, or pcDNA3.1-HA-STAT3 plasmid in a 24-well plate. The cells were washed with cold PBS buffer and lysed by passive lysis buffer after 24 h culture. The relative luciferase activities were then detected by a dual-luciferase reporter assay kit (Promega, USA) according to the product instructions on GloMax 20/20 luminometer. For the signaling pathway analysis, 7 luciferase reporter constructs (Genomeditech, Shanghai, China), including AP-1-luc, YAP/TAZ-luc, STAT3-luc, TCF/LEF-luc, SMAD2/3-luc, ARE-luc, and NF-κB-luc represent seven signaling pathways. These plasmids were respectively co-transfected with Renilla luciferase plasmid pRL-SV40 into UHMK1 knockdown cells and control cells in a 96-well plate, and measured the relative luciferase activities by dual-luciferase reporter assay kit as described above. All transfections were routinely performed in triplicate and the experiments were repeated three times.

### Chromatin immunoprecipitation (ChIP) assays

The ChIP assay was routinely performed with EZ ChIP Kits (Merk Milipore, USA). After cross-linked with formaldehyde, DLD-1 cells were subjected to ChIP assay following the manufacturer’s instructions. The immunoprecipitated DNA was amplified for the promoter region of UHMK1 and Cyclin D1 (CCND1) via PCR on a 2% agarose gel. The primers used were listed in Supplementary Table 1.

### Immunofluorescence staining

DLD-1/UHMK1-2 cells were seeded on coverslips for overnight with or without IL-6 and fixed with 4% paraformaldehyde for 10 min, followed by permeabilization with 0.1% Triton X-100 for 5 min. Cells were then rinsed three times with PBS and blocked by 10 % donkey serum for half an hour. After that, cells were incubated with FLAG tag antibody for UHMK1 (1:50) or STAT3 antibody (1:50) overnight. The Alexa Fluor 488 and AlexaFluor 594 secondary antibodies were then incubated for 1 h at room temperature. Coverslips were mounted on slides with DAPI. Cells were acquired and photographed on a confocal laser scan microscope (Zeiss, Germany).

### Animal model

BALB/c nude mice, 5 to 6-week-old, were purchased from Shanghai SLAC laboratory, China. 2 × 10^6^ of DLD-1 or RKO cells with UHMK1 overexpression or knockdown and their control cells were randomly subcutaneously injected into the dorsal flank of nude mice (for DLD-1 cells, *n* = 8 per group; for RKO cells, *n* = 6 per group), respectively. The mice were housed for 3 to 4 weeks in a specific-pathogen-free condition after injection. The tumors were measured each week by a digital caliper and the tumor volume was calculated using formula: 0.5 × large diameter × (small diameter)^2^. 3 to 4 weeks later, the mice were euthanized, and generated tumors were photographed and weighted. All the moue experiments and handling procedures were approved by the Institutional Animal Care and Use Committee of Shanghai East Hospital.

### Statistical analysis

The quantitative data used in this study are represented as mean ± standard deviation (SD) and analyzed by two-tailed student’s *t* test or χ2 test. Survival curves of CRC patients with UHMK1 high expression and low expression were calculated via the Kaplan–Meier method and determined by the log-rank test. A *P* value of <0.05 was considered as statistically significant difference.

## Results

### UHMK1 expression is upregulated in CRC

**Fig. 1:**
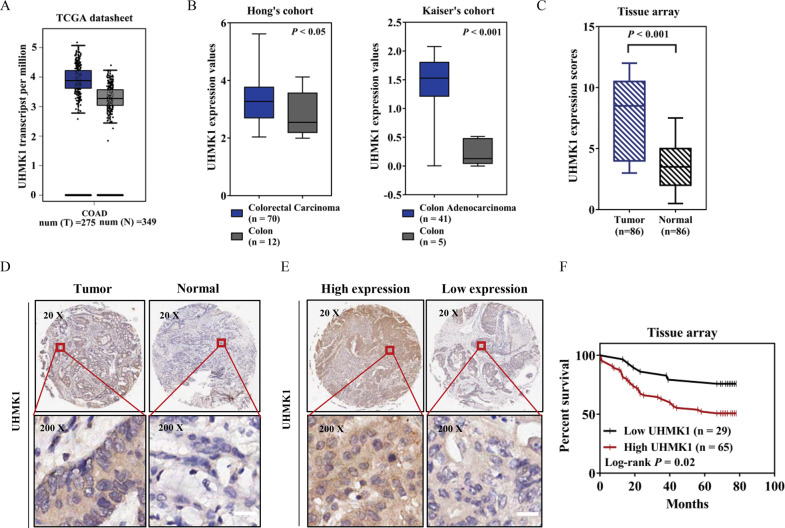
UHMK1 was overexpressed in CRC and significantly associated with poor prognosis. **A** UHMK1 mRNA level was higher in colon adenocarcinoma tissues than normal tissues (TCGA-COAD dataset, http://gepia.cancer-pku.cn/). **B** UHMK1 was upregulated in tumor tissues compared with normal tissues in colorectal carcinoma samples from Hong’cohort and colon adenocarcinoma tissues from Kaiser’s cohort. **C** Expression scores of UHMK1 in colon cancer tissues were higher than corresponding non-tumor tissues in a tissue microarray, *p* < 0.001. **D** Representative IHC images of UHMK1 in colon cancer tissues and adjacent normal colon tissues. Scale bar: 25 μm. **E** Representative IHC images indicating high expression or low expression of UHMK1 in colon cancer tissues. Scale bar: 25 μm. **F** Kaplan–Meyer plot of two groups of CRC patients classified by UHMK1 expression. Statistical analyses were performed with log-rank test (*P* = 0.02). Red, high expression group; black, low expression group.

According to TCGA-COAD (The Cancer Genome Atlas Colon Adenocarcinoma) data collection, UHMK1 mRNA expression was significantly higher in tumor tissue (*n* = 275) than normal tissue (*n* = 349) (Fig. 1A). Meanwhile, microarray dataset analyses from Hong’s dataset and Kaiser’s dataset indicated the upregulation of UHMK1 in colon adenocarcinoma tissues compared with normal controls [27, 28] (Fig. 1B). Subsequently, immunohistochemical analysis (IHC) was conducted to examine UHMK1 protein expression in a colon tissue microarray, which consists of 86 paired colon cancer and paracancerous tissues and eight cases of individual colon cancer specimens. Consistent with those online database analyses regarding mRNA, the protein expression of UHMK1 was also significantly increased in colon cancer tissues (Fig. 1C, D). Furthermore, we evaluated the relationship between UHMK1 expression and patients’ prognosis. The results showed that high expression of UHMK1 was correlated with decreased overall survival (Fig. 1E, F). These data strongly suggested that UHMK1 may play important roles in CRC.

### UHMK1 knockdown restrains CRC cell proliferation in vitro and tumorigenicity in vivo

**Fig. 2:**
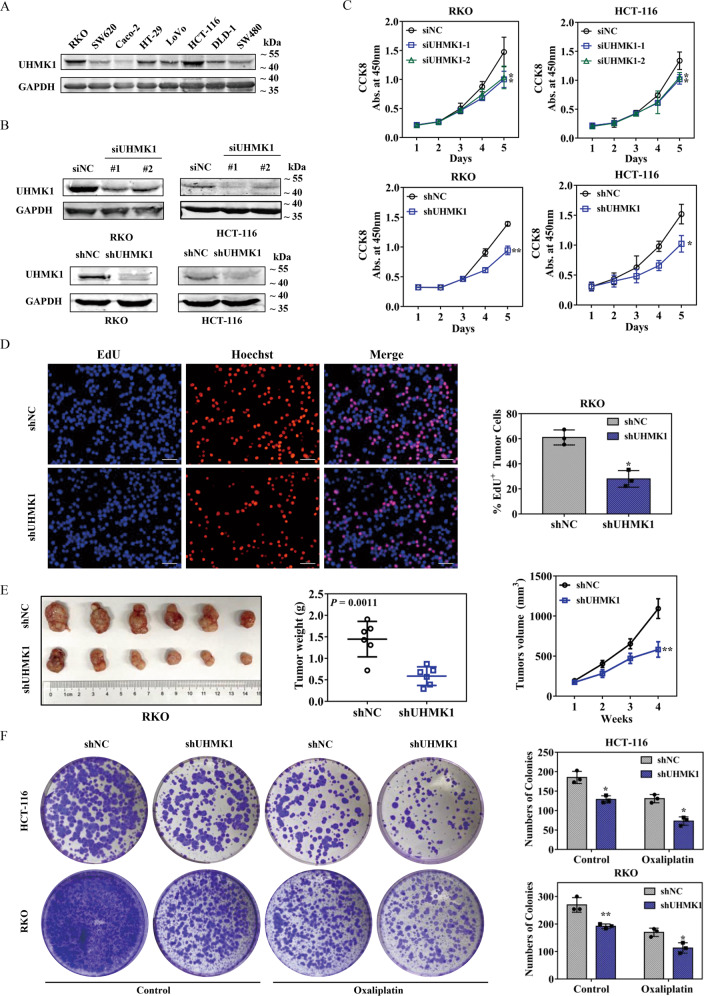
UHMK1 downregulation suppressed CRC cell proliferation, tumorigenecity and oxaliplatin resistance. **A** UHMK1 expression levels in different human CRC cell lines were determined by western blotting. **B** Western blot results of UHMK1 knockdown in RKO and HCT-116 cells. **C** CCK-8 analysis of UHMK1 knockdown in RKO and HCT-116 cells. **D** EdU staining was used to observe cell proliferation after UHMK1 knockdown in RKO cells. The number of positive EdU cells was recorded. Scale bar: 100 μm. **E** The image of tumors removed from nude mice implanted with RKO/shUHMK1 cells (*n* = 6) or normal control RKO cells (*n* = 6) for 4 weeks. Tumor weight and tumor volume were analyzed compared to the normal group. **F** Colony formation assay of UHMK1 knockdown in HCT-116 and RKO cells with or without oxaliplatin treatment (50 μM). *p < 0.05, **P < 0.01.

As demonstrated by western blotting in Fig. 2A, UHMK1 expression varied in CRC cell lines, both RKO and HCT116 showed high protein expression, while SW620, Caco-2, HT29, LoVo, DLD-1, and SW480 had relatively low UHMK1 expression. To explore the biological function of UHMK1 in CRC, a transient transfection model using siRNAs and a stable transfection model using lentivirus-mediated shRNA in RKO cells and HCT116 cells were established, which successfully downregulated the expression of UHMK1 (Fig. 2B). Then, the behaviors of RKO cells and HCT116 cells were examined by CCK-8 and EdU assays. The downregulation of UHMK1 significantly restrained the cell viability of both RKO cells and HCT116 cells compared to control groups in a 5-day period (Fig. 2C). In parallel, the ratio of EdU positive cells to the whole cells was decreased in the RKO-shUHMK1 group compared with the control group (Fig. 2D). The essential role of UHMK1 for cell proliferation was further confirmed in subcutaneous xenograft tumor models. As shown in Fig. 2E, the tumorigenicity of RKO/shUNMK1 cells was significantly attenuated since lower tumor weight and smaller tumor volume were observed after 4 weeks. In addition, UHMK1 knockdown exhibited a resistance to oxaliplatin as demonstrated in the colony formation assays (Fig. 2F). These findings revealed that UHMK1 silencing hinders proliferation and oxaliplatin resistance of CRC cells.

### UHMK1 overexpression promotes CRC cell proliferation and oxaliplatin resistance

**Fig. 3:**
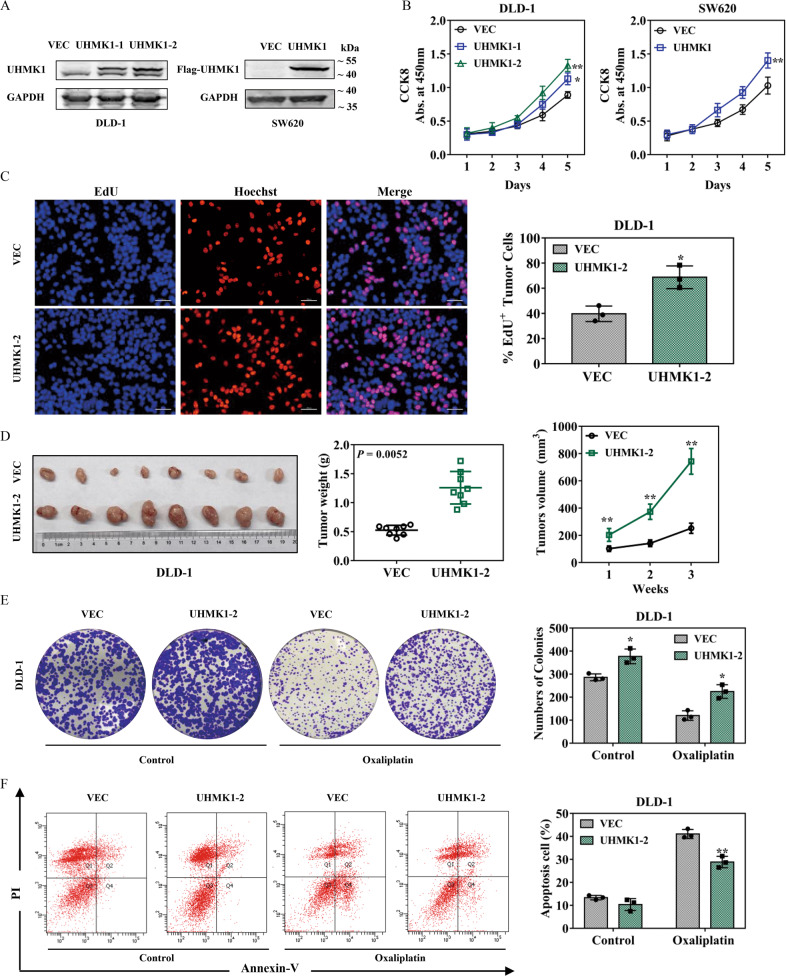
The effects of UHMK1 upregulation on CRC cell proliferation, tumorigenicity and oxaliplatin resistance. **A** Western blot results of UHMK1 overexpression in DLD-1 and SW620 cells. Anti-UHMK1 (left) and anti-FLAG (right) antibodies were used. **B** The growth rates of DLD-1 and SW620 cells with or without UHMK1 overexpression were monitored by CCK-8 assay. **C** EdU staining was used to detect cell proliferation in DLD-1 cells with UHMK1 stable overexpression and control cells. The number of positive EdU cells was recorded. Scale bar: 100 μm. **D** The image of tumors removed from nude mice implanted with DLD-1/UHMK1-2 cells (*n* = 8) and control RKO cells (*n* = 8) for 3 weeks. Tumor weight and tumor volume were analyzed compared to control group. **E** Colony formation assays in UHMK1 stably overexpressed DLD-1 cells and control cells with or without oxaliplatin treatment (100 μM). **F** FACS analysis of apoptotic DLD-1 cells with or without oxaliplatin treatment (300 μM). UHMK1 stable expression cells had an anti-apoptosis effect. *p < 0.05, **P < 0.01.

To verify the assumption made above, two stable UHMK1 overexpressed CRC cell lines (DLD-1/UHMK1-1 and -2, SW620/UHMK1) were established by serial dilution method (Fig. 3A). CCK-8 assays were then employed, and the results indicated that overexpression of UHMK1 promoted the proliferation of DLD-1 cells and SW620 cells (Fig. 3B). Consistently, EdU assay and in vivo subcutaneous tumor xenograft assay also confirmed that UHMK1 overexpression promoted CRC cell proliferation and played a crucial role in tumor growth (Fig. 3C, D). To test whether UHMK1 overexpression influences oxaliplatin resistance, DLD-1/UHMK1-2 cells were then treated with oxaliplatin followed by colony formation assay. The results showed that overexpression of UHMK1 enhanced the resistance of DLD-1 cells to oxaliplatin (Fig. 3E). Flow cytometric analysis was used to investigate whether UHMK1 overexpression affects oxaliplatin-induced apoptosis. Compared with the control group, DLD-1/UHMK1-2 cells showed a significantly decreased rate of apoptosis upon treatment with oxaliplatin (Fig. 3F), implicating that the oxaliplatin-induced apoptosis was weakened by overexpression of UHMK1.

### UHMK1 affects JAK/STAT3 signaling pathway by interacting with STAT3

**Fig. 4:**
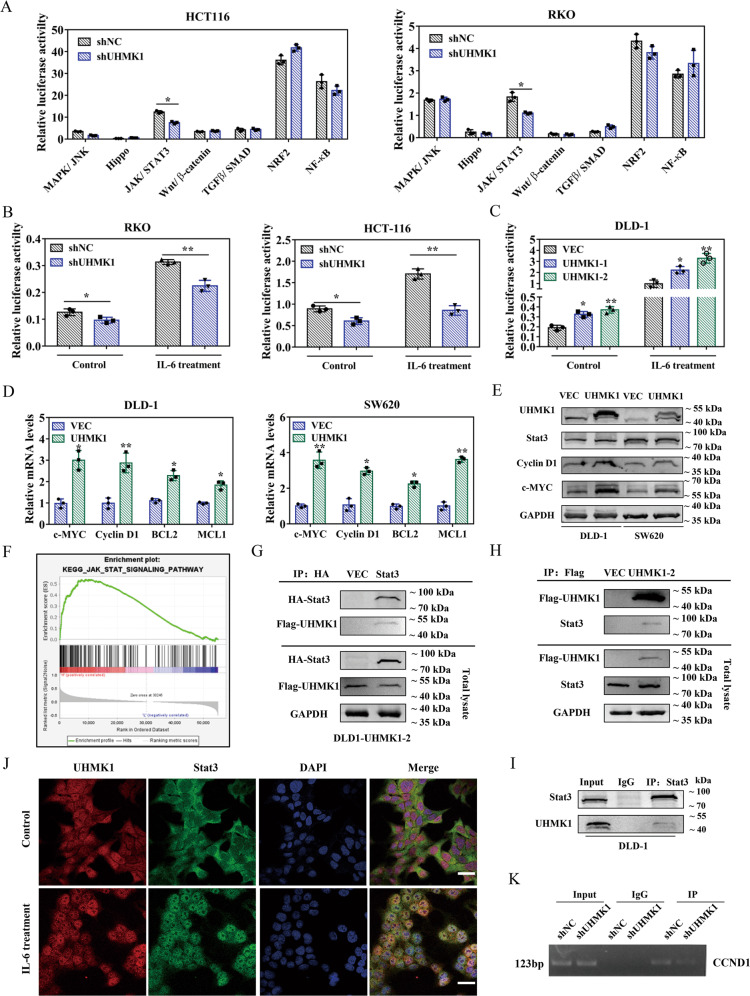
UHMK1 was involved in the regulation of JAK/STAT3 pathway. **A** Dual-luciferase reporter assays were performed in HCT116/shUHMK1 and RKO/shUHMK1 cells with seven typical signaling pathway reporter systems as indicated. **B** Dual-luciferase reporter assay for JAK/STAT3 pathway in HCT116/shUHMK1 and RKO/shUHMK1 cells after IL-6 treatment. **C** Dual-luciferase reporter assay for JAK/STAT3 pathway in DLD-1/UHMK1-1 and DLD-1/UHMK1-2 cells after IL-6 treatment. **D** Relative mRNA expression of c-MYC, Cyclin D1, BCL2, and MCL1 in DLD-1/UHMK1 and SW620/UHMK1 cells by RT-qPCR. **E** Western blot results of UHMK1, Cyclin D1, and c-MYC expression in DLD-1/UHMK1 and SW620/UHMK1 cells. **F** GSEA analysis comparing the gene sets of JAK/STAT pathway with UHMK1 expression. Data were obtained from TCGA database. **G** Co-IP assays were performed in DLD-1/UHMK1-2 cells transiently transfected with HA-STAT3 plasmid. Exogenous UHMK1 was immunoprecipitated with the anti-HA antibody. **H** Endogenous STAT3 was pulled down with anti-FLAG antibody in DLD-1/UHMK1-2 cells via Co-IP analysis. **I** Endogenous UHMK1 was coimmunoprecipitated with anti-STAT3 antibody in DLD-1 cells. **J** Co-localization of both UHMK1 (red) and STAT3 (green) in DLD-1/UHMK1-2 cells was shown. Scale bar: 30 μm. **K** Results of ChIP assay conducted using chromatins isolated from RKO cells with UHMK1 knockdown and control cells. The immunoprecipitated DNA by anti-STAT3 antibody was analyzed by agarose gel electrophoresis. Normal IgG was used as a negative control. *p < 0.05, **P < 0.01.

To explore the mechanism by which UHMK1 promotes cell proliferation and oxaliplatin resistance, dual-luciferase reporter assays were performed in HCT116 and RKO cells. Among seven signaling pathways, only JAK/STAT3 pathway was significantly influenced and exhibited reduced relative luciferase activity after UHMK1 knockdown (Fig. 4A), suggesting a possible involvement of UHMK1 in the JAK/STAT3 pathway. For further investigations, IL-6 was used as a JAK/STAT3 pathway inducer to magnify the luciferase activity. As shown in Fig. 4B, UHMK1 knockdown strongly decreased the activation of the JAK/STAT3 pathway induced by IL-6, whereas overexpression of UHMK1 increased the pathway activation (Fig. 4C). Next, we examined the expression of four main downstream targets of this pathway, c-MYC, Cyclin D1, BCL2, and MCL1 using qRT-PCR. Compared to the control group, a significant upregulation of the four factors was found when UHMK1 was overexpressed (Fig. 4D). Similar results were observed for c-Myc and Cyclin D1 protein expression in UHMK1 overexpressed DLD-1 and SW620 cells (Fig. 4E). In the meantime, Gene Set Enrichment Analysis (GSEA) was performed using TCGA-COAD datasets and verified the positive correlation of UHMK1 with JAK/STAT3 signaling (Fig. 4F).

To gain more insights into the regulation of STAT3 signaling by UHMK1, we detected the expression and nucleus-cytoplasm translocation of STAT3 when UHMK1 was silenced or overexpressed. As a result, the total and phosphorylated STAT3 protein levels were not significantly changed with UHMK1 expression alteration (Supplementary Fig. 1A, B), nor the nuclear localization of STAT3 (Supplementary Fig. 1C). Finally, we examined whether the two proteins have an interaction by co-immunoprecipitation experiments. Exogenous FLAG-tagged UHMK1 was pulled down with HA-tagged STAT3, and likewise STAT3 was detected in FLAG-UHMK1 immunoprecipitated complex (Fig. 4G, H). Remarkably, UHMK1 was also seen in endogenous immunoprecipitated STAT3 complex (Fig. 4I). According to the literature reported previously [29], we constructed a mutant form of STAT3 which has defective in DNA binding (STAT3-mut). The data from Co-IP assay showed that STAT3-mut still had an interaction with UHMK1, which is similar to the STAT3-wt (Supplementary Fig. 2A), indicating that STAT3 mutation in the DNA binding sites did not affect the interaction with UHMK1. Confocal assays further demonstrated that the two proteins had a co-localization in DLD-1 cells (Fig. 4J). These data suggested that UHMK1 and STAT3 could interact with each other. More importantly, ChIP assays showed that UHMK1 downregulation decreased the binding of STAT3 to the Cyclin D1 promoter region that was proved by previous reports as STAT3 binding site [30, 31] (Fig. 4K). Taken together, these findings implicated that UMHK1 is involved in the regulation of JAK/STAT3 signaling and affects STAT3 activity by interaction with STAT3.

### STAT3 transcriptionally regulates UHMK1 expression in CRC cells

**Fig. 5:**
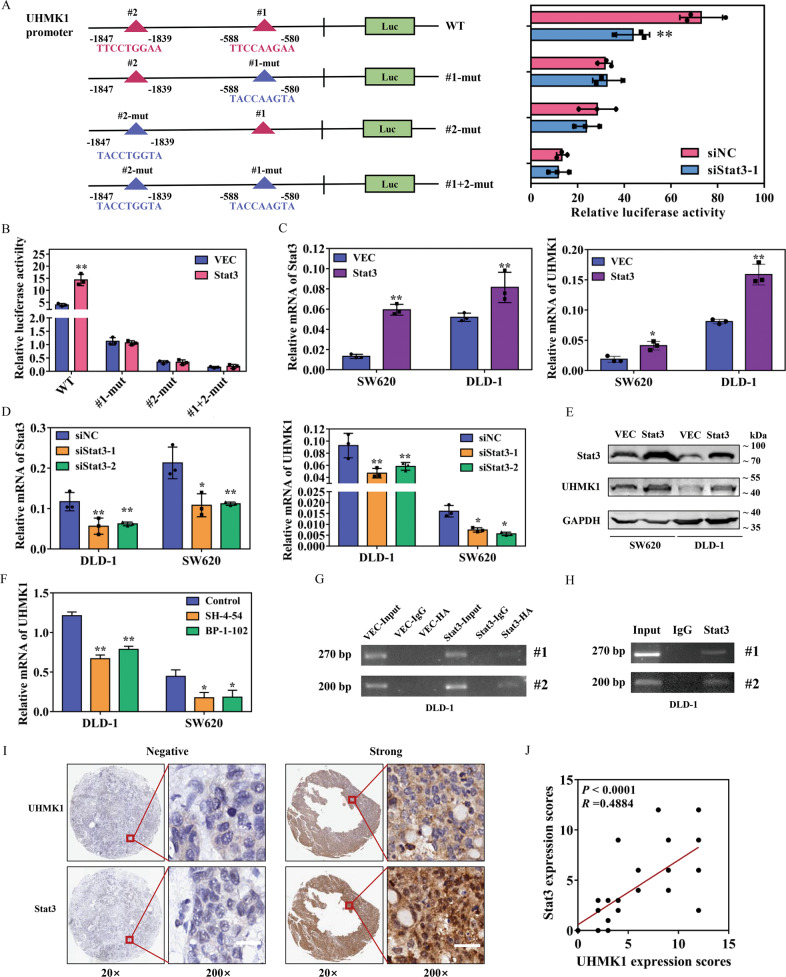
UHMK1 was transcriptionally regulated by STAT3. **A** Two putative consensus-binding sites of STAT3 were shown in the UHMK1 promoter region (−1847 to −1839 bp, −588 to −580 bp). Wild-type or mutant UHMK1 promoter reporter plasmids were co-transfected with si-STAT3 or si-NC into 293 T cells. Then the promoter activity was examined by dual luciferase assay. **B** Relative luciferase activity of the UHMK1 promoter reporters in 293 T cells when STAT3 was overexpressed. **C** The relative mRNA levels of STAT3 and UHMK1 in SW620 and DLD-1 cells transfected with STAT3 plasmid. **D** The relative mRNA expression of STAT3 and UHMK1 in STAT3 knockdown cells and control cells. **E** The protein expression of UHMK1 was examined by western blotting when STAT3 was upregulated. **F** The mRNA expression of UHMK1 was detected after treatment with the two STAT3 inhibitors: SH-4-54 or BP-1-102 (10 µM, 24 h for each). **G** ChIP assays were employed using chromatins isolated from DLD-1 cells transfected with STAT3 or empty vector. The immunoprecipitated DNA by anti-HA antibody was analyzed by agarose gel electrophoresis. **H** ChIP assay was performed with endogenous STAT3 protein. Normal IgG was used as a negative control. **I** Representative images of immunohistochemical staining of CRC tissue sections from 30 cases using UHMK1 antibody and STAT3 antibody (20x magnification in the main images, 200x magnification in the inserts). Scale bar: 30 μm. **J** Assessment of the correlation between UHMK1 and STAT3 expression in CRC specimens (*n* = 30) using Pearson correlation coefficient analysis. Some of the dots on the graph represent more than one specimen. *p < 0.05, **P < 0.01.

Two putative consensus binding sites of STAT3 (5’-TTCNNNGAA-3’) were found on UHMK1 promoter region located at −1847 and −588bp upstream of the transcriptional starting site. To assess whether STAT3 regulates UHMK1 expression, we constructed the wild-type promoter and site-mutated promoter of UHMK1 into pGL3-basic vector, respectively. As a result of dual luciferase reporter assays, the disruption of the STAT3 binding site significantly attenuated UHMK1 promoter activity in 293 T cells, especially when both sites were mutated (Fig. 5A). The concordant results could also be seen in DLD-1 cells that STAT3 overexpression had no effect on mutated UHMK1 promoter activity (Fig. 5B) Moreover, qRT-PCR and western blot results showed that UHMK1 expression levels were changed with STAT3 knockdown or overexpression (Fig. 5C–E). Meanwhile, SH-4-54 and BP-1-102 as STAT3 inhibitors markedly abolished UHMK1 expression in DLD-1 and SW620 cells (Fig. 5F). These data suggested that UHMK1 is regulated by STAT3 at mRNA level. To provide more direct evidence, we conducted a ChIP assay using chromatins prepared from DLD-1 cells. The results indicated that exogenous HA-tagged STAT3 or endogenous STAT3 bound to both putative binding sites (Fig. 5G, H). In addition, a positive association between STAT3 and UHMK1 expression was observed in a 30 cases of tissue microarray by immunostaining with UHMK1 antibody and STAT3 antibody (Fig. 5I, J). Collectively, these data strongly indicated that STAT3 can transcriptionally regulate UHMK1 expression.

### STAT3 knockdown decreases UHMK1-induced cell proliferation and chemo-resistance and vice versa

**Fig. 6:**
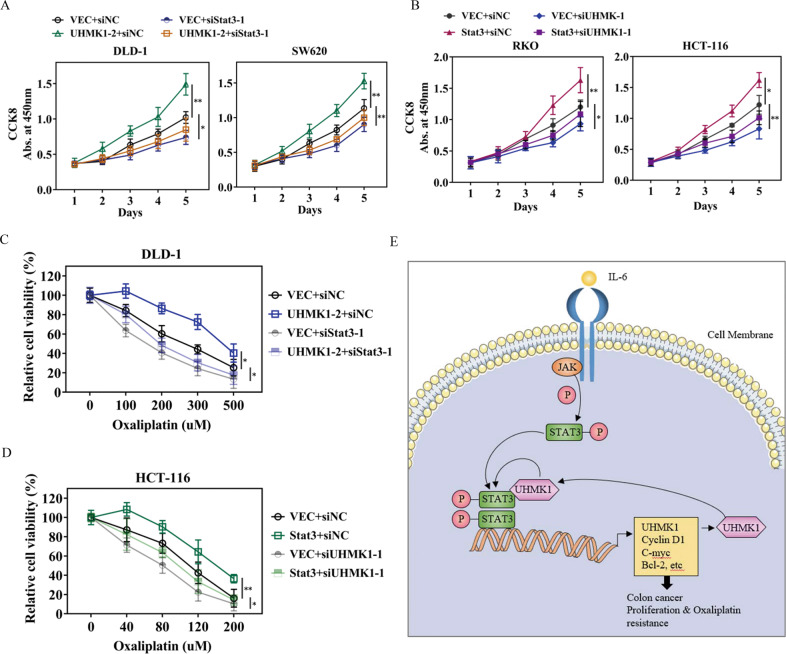
The functional relationship between UHMK1 and STAT3 in regulating cell proliferation and oxaliplatin resistance. **A** Cell viability was measured by CCK-8 assay in DLD-1/UHMK1-2 or SW620/UHMK1 cells with or without STAT3 knockdown. **B** CCK-8 assay in RKO and HCT-116 cells transfected with si-NC, siUHMK1-1, empty vector or STAT3 plasmid in a combined way as indicated. **C** Cell viability was measured by CCK-8 under gradient oxaliplatin treatment using cells as indicated in (**A**). **D** Cell viability was measured by CCK-8 under gradient oxaliplatin treatment using cells as indicated in (**B**). *p < 0.05, **P < 0.01. **E** Schematic diagram of the positive feedback loop between UHMK1 and STAT3.

To determine the functional relationship between UHMK1 and STAT3 in CRC cell proliferation and chemoresistance, we silenced STAT3 in UHMK1 overexpressed DLD1 and RKO cells. The CCK-8 assays revealed that STAT3 silencing decreased UHMK1 overexpression induced cell viability under normal culture conditions or oxaliplatin treated conditions (Fig. 6A, C). As well, UHMK1 knockdown in STAT3 overexpressed cells also retarded STAT3 mediated cell growth and oxaliplatin resistance (Fig. 6B, D). These observations implicated that UHMK1 and STAT3 interdependently regulate cell proliferation and respond to chemotherapy. In addition, the results from CCK-8 assays revealed that STAT3-mut had no effect on RKO cell viability compared to STAT3-wt with or without UHMK1 overexpression (Supplementary Fig. 2B), suggesting that STAT3 transcription activity is required for itself or UMHK1 induced cell growth. Thus, together with previous findings, we propose a UHMK1/STAT3 positive feedback loop involved in the regulation of CRC cell proliferation and chemoresistance, as depicted in Fig. 6E.

## Discussion

UHMK1, also named KIS, was previously reported as a kinase interacting with Stathmin, which is involved in the regulation of microtubule dynamics [32]. Emerging evidence has indicated UHMK1 to be an essential factor in many kinds of tumors, including liver cancer, ovarian cancer, and gastric cancer [21, 23, 24]. In the present study, we demonstrated that UHMK1 was significantly upregulated in CRC cancer tissues, and high expression of UHMK1 was related to poor overall survival, although we did not find its association with other clinicopathological characteristics of this cancer. Moreover, we proved for the first time that UHMK1 downregulation restrained CRC cell proliferation and tumor growth. On the contrary, UHMK1 upregulation significantly promoted CRC cell line proliferation and in vivo tumor growth. Also, UHMK1 expression was positively related to the oxaliplatin resistance of CRC cells, implying that UHMK1 could be an anti-apoptotic factor. Given the clinical and biological significance of the UHMK1, our study indicated that UHMK1 could be a potential biomarker for CRC prognosis and could have potential utilization in CRC treatment in the future.

Mechanistically, Teng et al. described UHMK1 as a novel target gene of YAP/FOXM1, stimulates nuclear enrichment of oncogene MYBL2, and supports liver cancer cell proliferation [23]. Chu et al. reported that COX5B-UHMK1-ERK axis promotes hepatoma cell growth and migration [22]. UHMK1 also contributes to purine metabolism reprogramming by regulating the NCOA3/ATF4 axis and significantly promotes gastric cancer development [21]. A recent study showed that EBLN3P promotes tumor development via targeting miR-323a-3p/UHMK1 in CRC [33]. In addition, UHMK1 expression is under the control of oxidative phosphorylation [22]. It could be a potential biomarker for dysregulation of oxidative phosphorylation. In the present study, we found a new signaling pathway that UHMK1 may be involved during carcinogenesis. Through dual-luciferase reporter assay screening, we discovered that UHMK1 expression was highly related to JAK/STAT3 signaling. Downregulation of UHMK1 strongly attenuated the activity of this signaling, implicating the involvement of UHMK1 in the pathway. On the other hand, we also provided evidence that UHMK1 was regulated by STAT3. By promoter sequence analysis, we observed two putative binding sites of STAT3 in the UHMK1 promoter. ChIP assays confirmed the binding of STAT3 to the putative promoter region. qRT-PCR and western blot data supported the speculation that STAT3 can transcriptionally increase UHMK1 expression. Thus, we are the first to propose a new UHMK1/STAT3 positive feedback loop to augment JAK/STAT3 signaling and lead to cancer development and progression.

A considerable amount of literature has demonstrated that JAK/STAT3 pathway is extensively involved in tumor progression [4, 34, 35]. In CRC, the JAK/STAT3 pathway can directly regulate tumor immune cells and upregulates the expression of oncogenic proteins, such as c-MYC, Cyclin D1, BCL2 or MCL1 to help drive tumor progression and chemoresistance [36]. In terms of this, our results revealed that UHMK1 may reinforce the binding of STAT3 to downstream targets via their protein interaction. However, the exact mechanisms of how UHMK1 interacts with STAT3 are still unknown. It has been reported in the literature from Ng et al. that STAT3 can be directly associated with Stathmin, which is known as an oncogenic protein in metastatic colorectal cancer [37, 38]. Interestingly, UHMK1 also interacts with Stathmin as reported previously [32]. Thus, there might exist a kind of possibility that UHMK1 and STAT3 can tie together via Stathmin as the media. This question should be classified in the future.

In conclusion, our findings demonstrate that UHMK1 is overexpressed in CRC, as in other cancer types. Its upregulation is closely correlated with poor overall survival in CRC patients. UHMK1 could promote cell proliferation and enhance oxaliplatin resistance via augmenting IL-6/JAK/STAT3 signaling. Our data provide new insights into the regulation of STAT3 signaling in the development of CRC. UHMK1 could be a potential therapy target for this disease.

## Supplementary information

related manuscript

Supplementary Table 1

Supplementary Fig.1

Supplementary Fig.2

Supplementary legend

Original Data File

aj-checklist

## Data Availability

All data analyzed and generated in this study are included in this published article and its supplementary information files.
