# Supplementary material for: UHMK1 aids colorectal cancer cell proliferation and chemoresistance through augmenting IL-6/STAT3 signaling
Source: Cell Death Dis. 2022 May 2;13(5):424. doi: 10.1038/s41419-022-04877-8 (PMC9061793; doi:10.1038/s41419-022-04877-8)
Supplement: Supplementary file 2 — Supplementary Table 1 [file 41419_2022_4877_MOESM2_ESM.docx]

Supplementary Table 1 Primers for qRT-PCR used in this study

|  | Forward Primer | Reverse Primer |
| --- | --- | --- |
| UHMK1 | AGAGAAACCATGGGCAGAAG | CAAGCCATGAAACAGCATCT |
| ACTIN | CCTGGCACCCAGCACAATG | GGGCCGGACTCGTCATACT |
| CCND1 | GGATGCTGGAGGTCTGCGA | TAGAGGCCACGAACATGCAAGT |
| c-MYC | TCTGGATCACCTTCTGCTGG | AGGATAGTCCTTCCGAGTGG |
| STAT3 | CAGGAGGGCAGTTTGAGTCC | CAAAGATAGCAGAAGTAGGAGA |
| BCL2 | ACTGGCTCTGTCTGAGTAAG | CCTGATGCTCTGGGTAAC |
| MCL1 | CGGTAATCGGACTCAACCTC | CCTCCTTCTCCGTAGCCAA |

Primers for ChIP assay to amplify promoter sequence of UHMK1 and Cyclin D1

|  | Forward Primer | Reverse Primer |
| --- | --- | --- |
| UHMK1-1# | AGAGGTCACCTTCACCACCA | TGTGAGGAGTCACAGCCTAC |
| UHMK1-2# | TCCTTCCAGGTTATAGGTAG | GGTCAACACAGACCTTCGGT |
| CCND1 | CCGGGCTTTGATCTTTGCT | GACTCTGCTGCTCGCTGCTA |
