# Supplementary figures and images for: UHMK1 aids colorectal cancer cell proliferation and chemoresistance through augmenting IL-6/STAT3 signaling
Source: Cell Death Dis. 2022 May 2;13(5):424. doi: 10.1038/s41419-022-04877-8 (PMC9061793; doi:10.1038/s41419-022-04877-8)

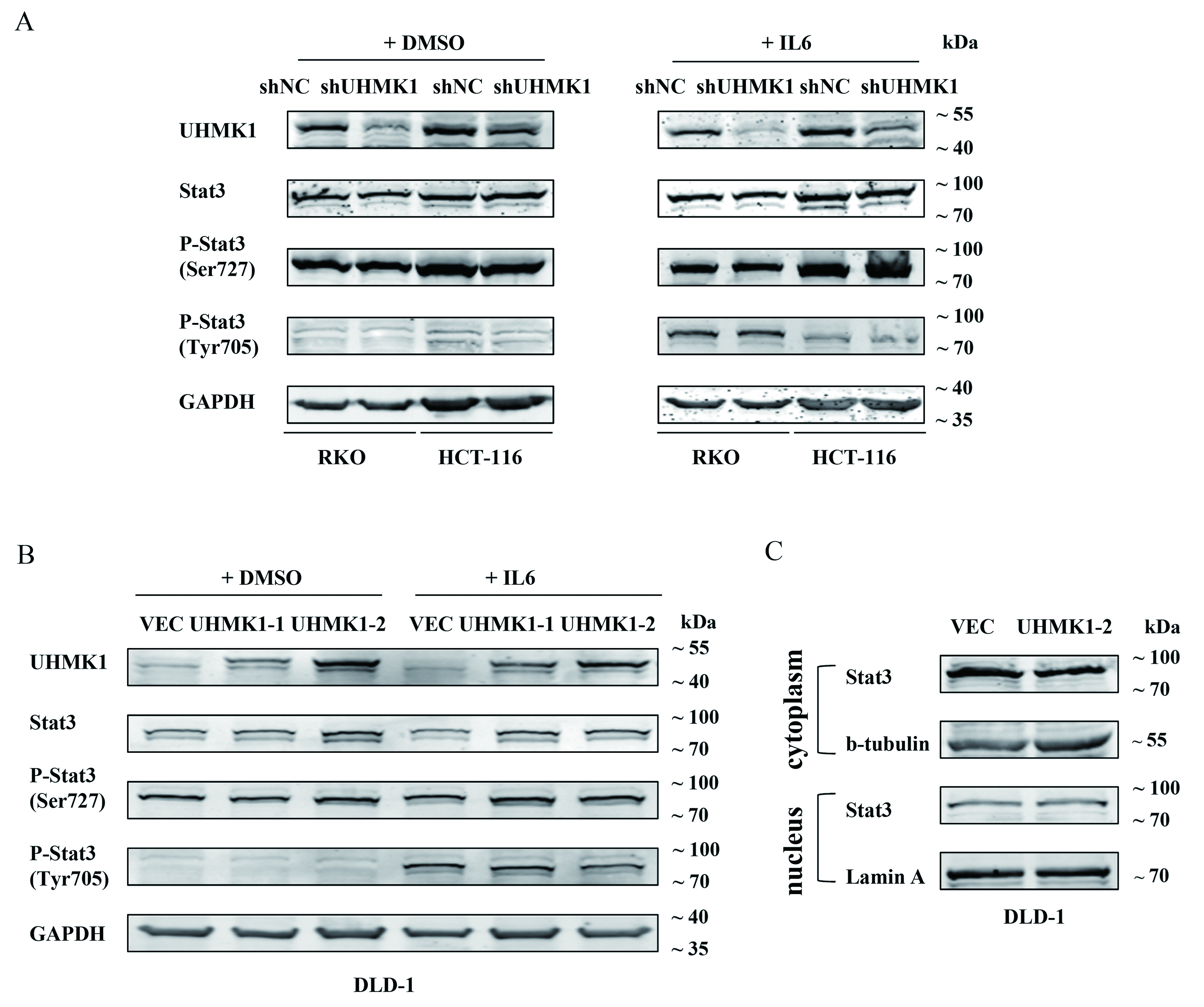

Supplement: Supplementary file 3 — Supplementary Fig.1 [file 41419_2022_4877_MOESM3_ESM.tif]

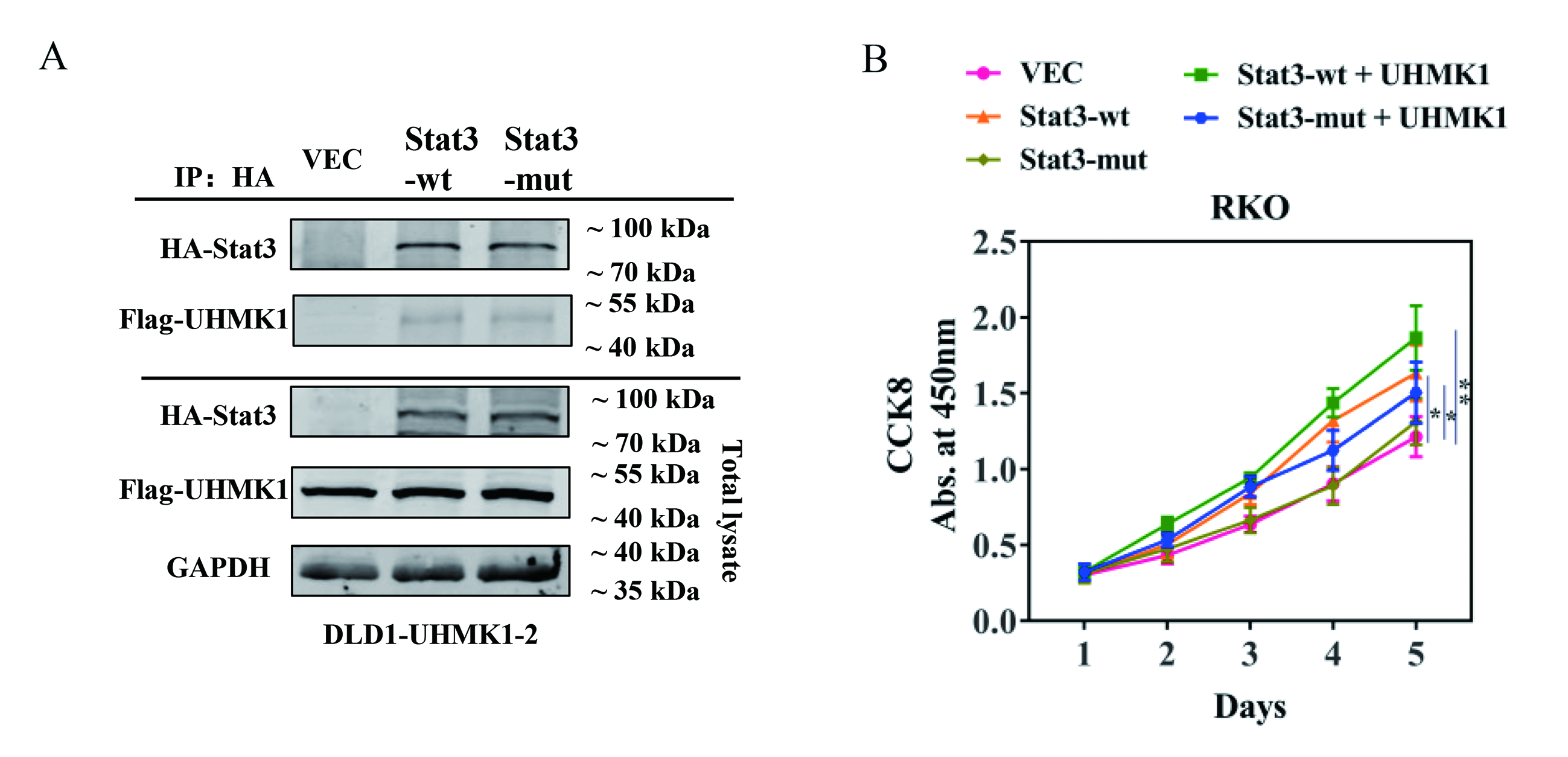

Supplement: Supplementary file 4 — Supplementary Fig.2 [file 41419_2022_4877_MOESM4_ESM.tif]

Fig.2

A

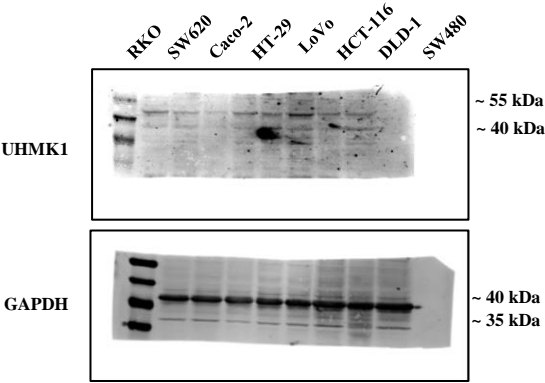

B

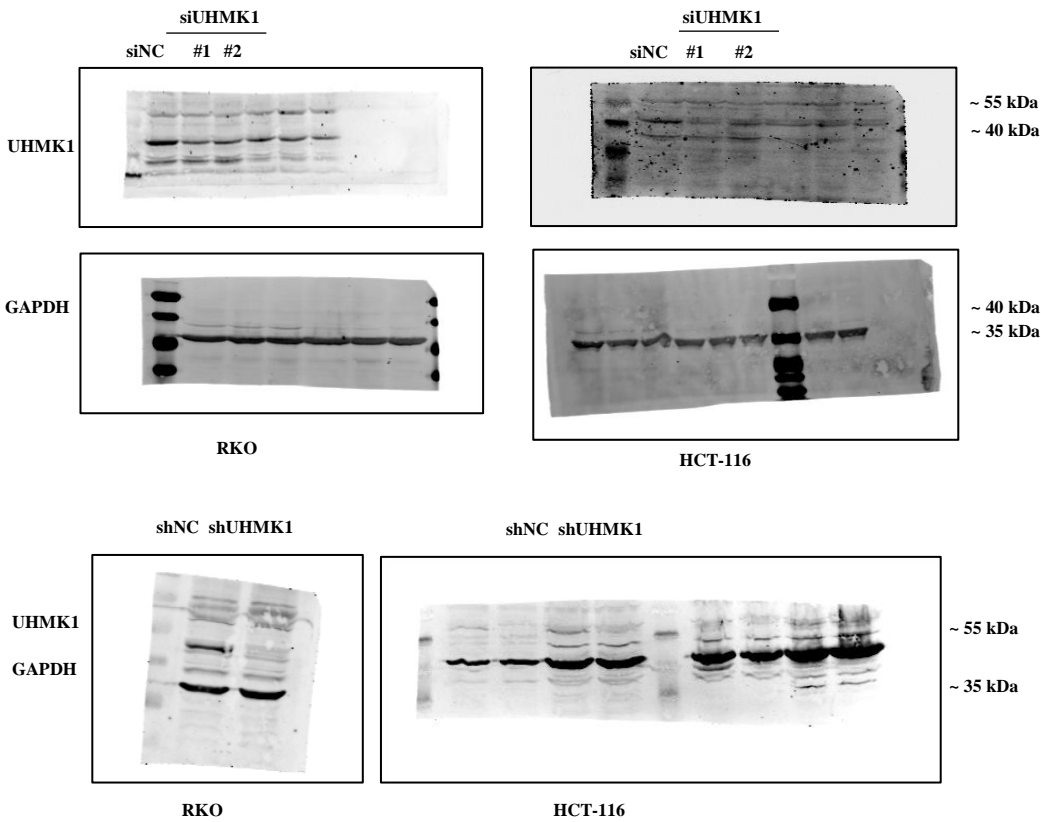

Fig.3

A

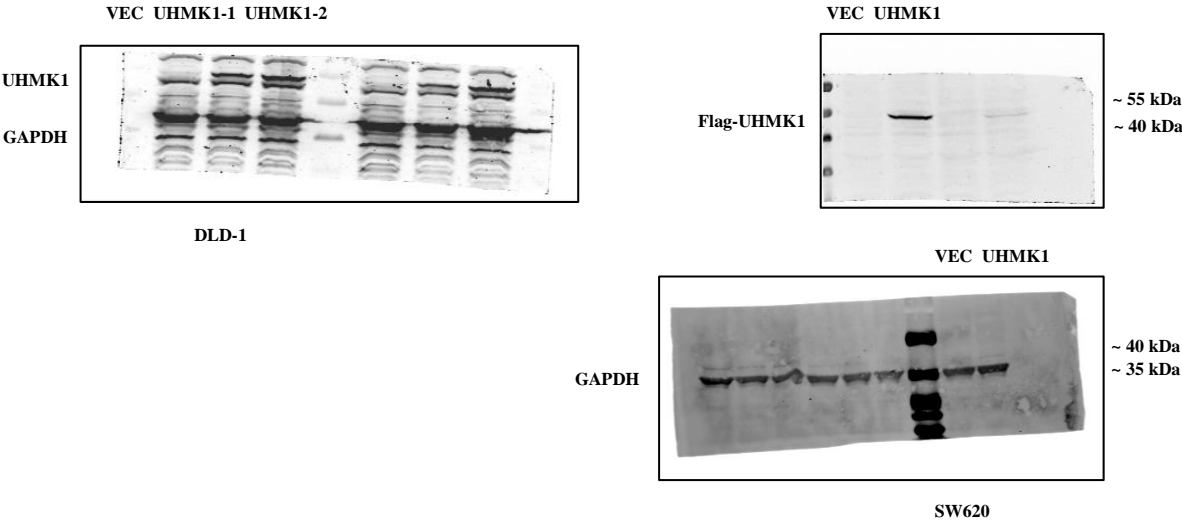

Fig.4

E

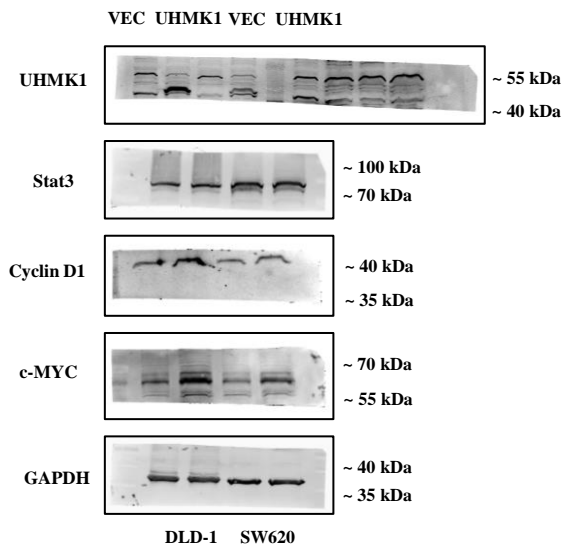

G

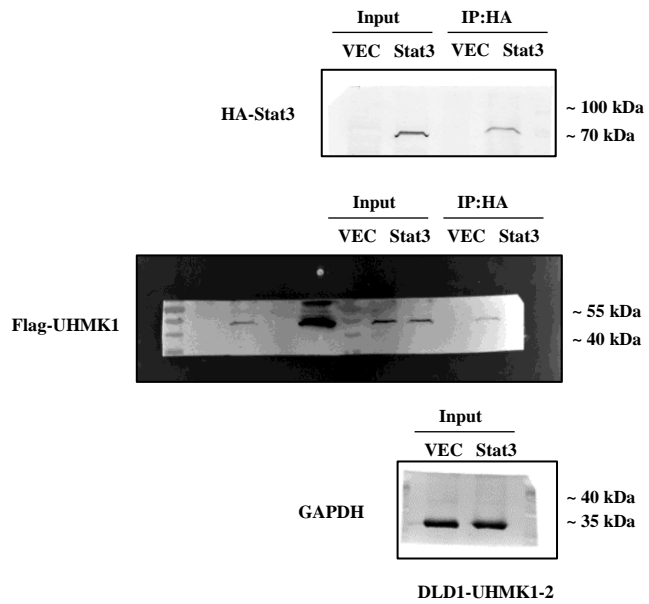

H

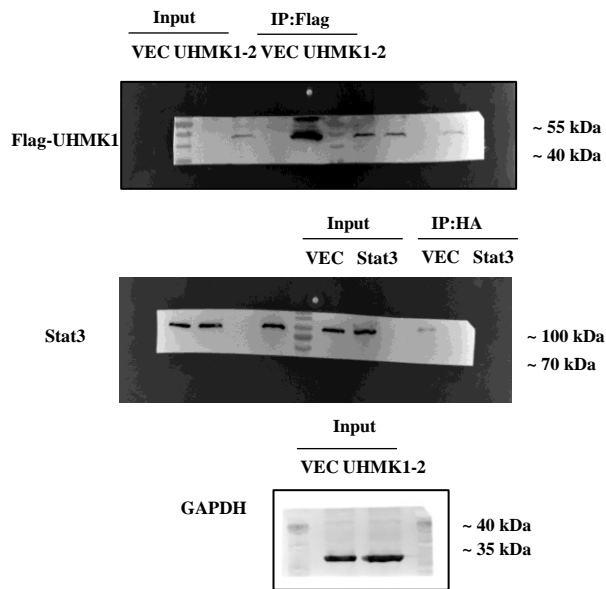

I

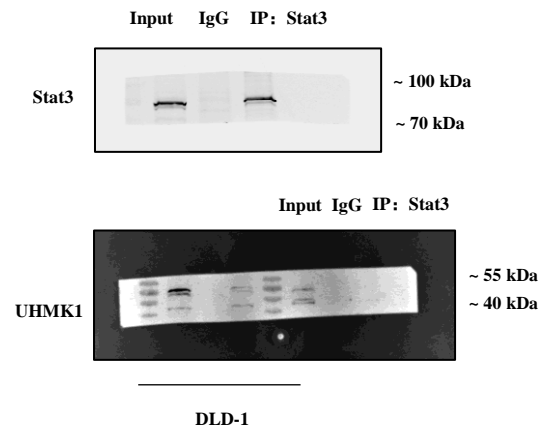

Fig.5

E

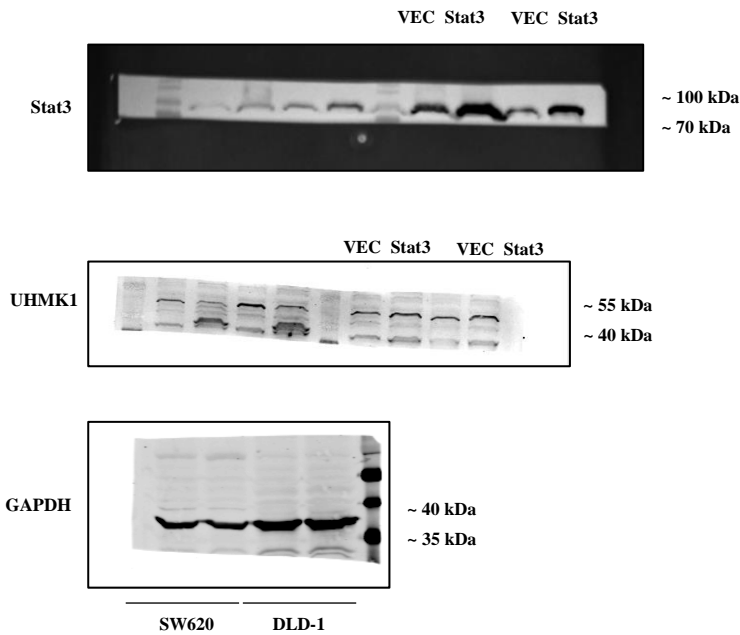

## Supplementary Figure 1

A

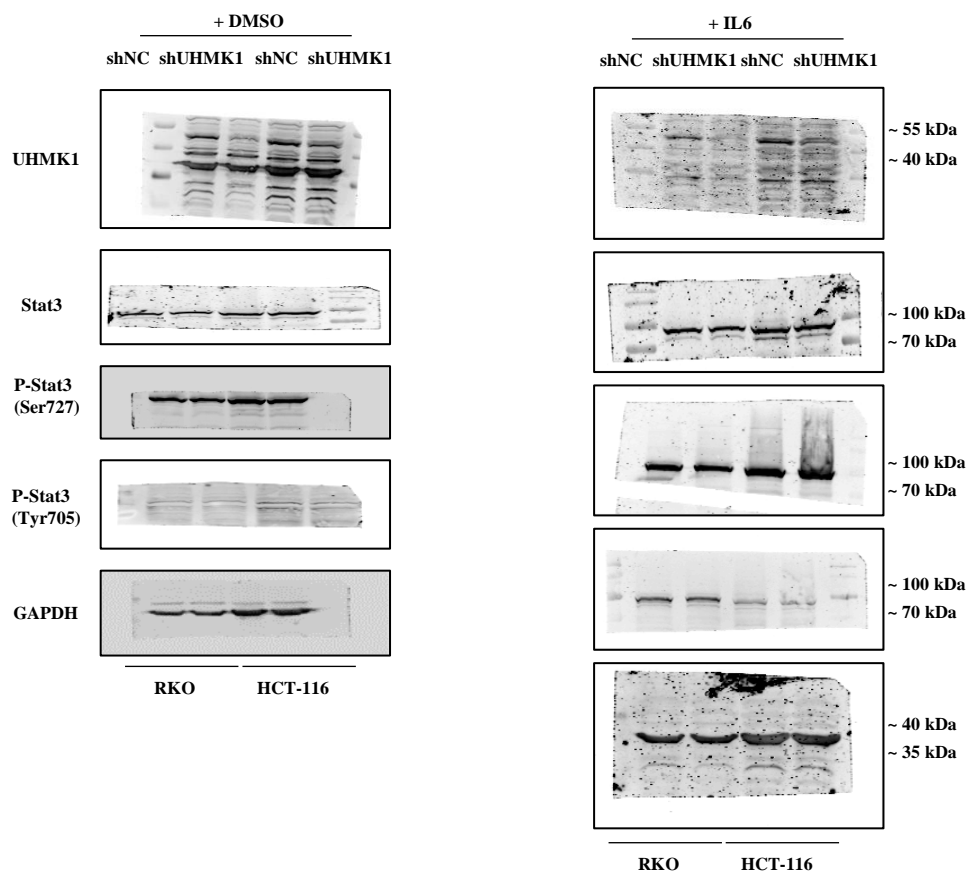

B

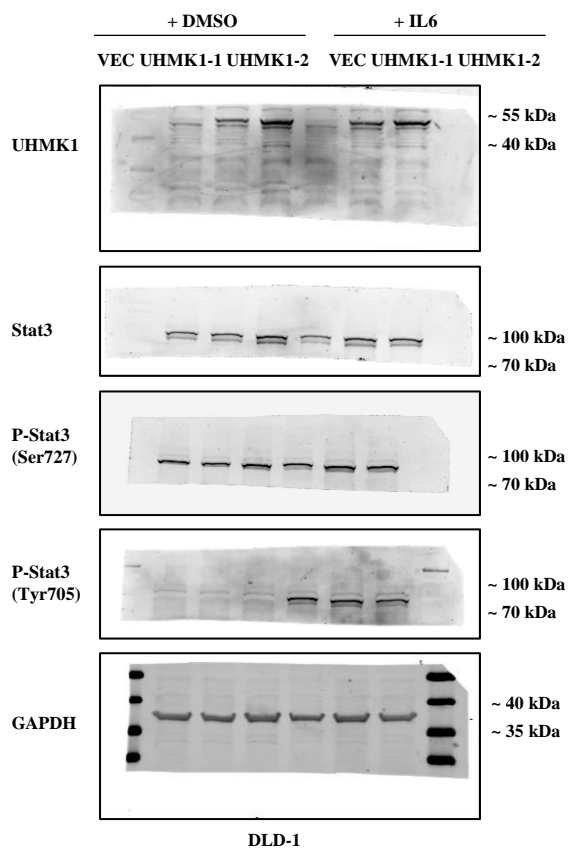

C

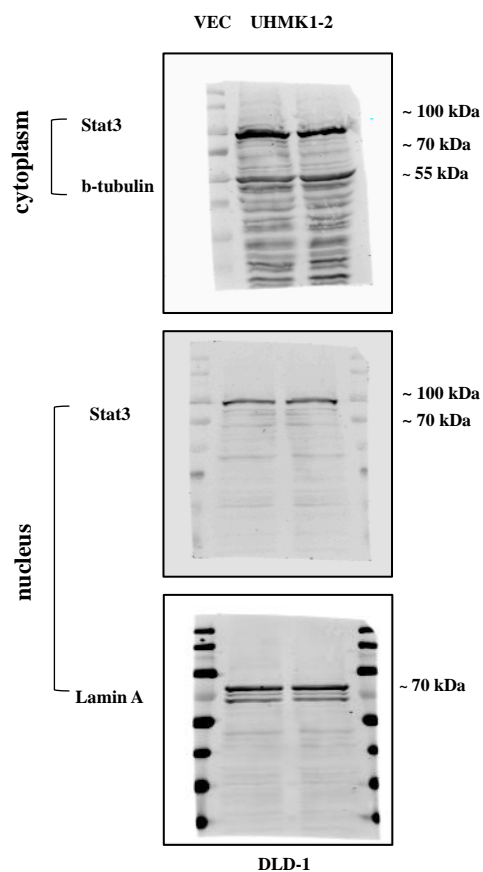

Supplementary Figure 2

A

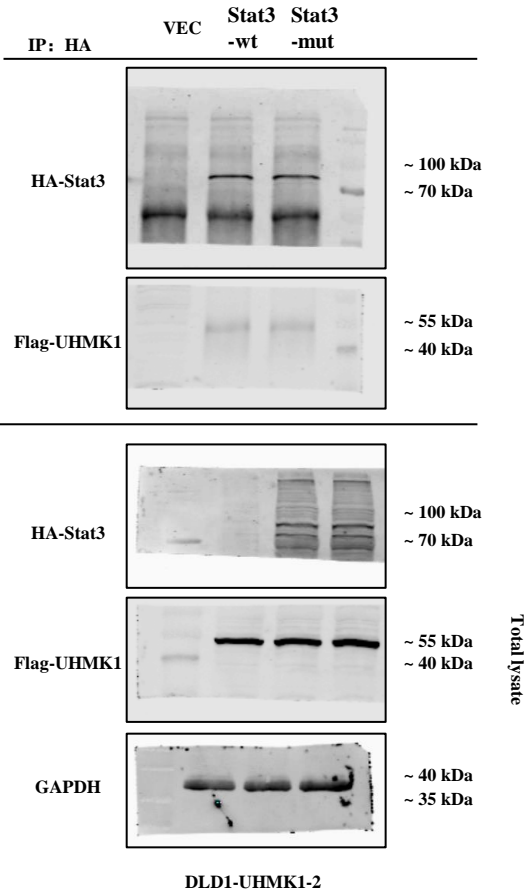

Supplement: Supplementary file 6 — Original Data File [file 41419_2022_4877_MOESM6_ESM.pdf]
