# Supplementary material for: UHMK1 aids colorectal cancer cell proliferation and chemoresistance through augmenting IL-6/STAT3 signaling
Source: Cell Death Dis. 2022 May 2;13(5):424. doi: 10.1038/s41419-022-04877-8 (PMC9061793; doi:10.1038/s41419-022-04877-8)
Supplement: Supplementary file 5 — Supplementary legend [file 41419_2022_4877_MOESM5_ESM.docx]

**Supplementary Fig.1. The regulation of STAT3 signaling in CRC cells by UHMK1.**  **(A)** Western blot results of UHMK1, STAT3, and two p-STAT3 expressions (Ser727 and Tyr705) with or without IL-6 induction in RKO and HCT-116 cells. **(B)** Western blot results of UHMK1, STAT3, and two p-STAT3 expressions (Ser727 and Tyr705) with or without IL-6 induction in DLD-1 cells. **(C)** Western blot result of STAT3 expression in cytoplasm and nucleus in control DLD-1 cells and DLD-1/UHMK1-2 cells. P-STAT3: phosphorylated STAT3.

**Supplementary Fig.2. STAT3 mutant defective in DNA binding has no evident effect on the association of STAT3 with UHMK1 and UHMK1 induced cell proliferation. (A)** Co-IP assays were performed in DLD-1/UHMK1-2 cells transiently transfected with STAT3-wt or STAT3-mut plasmid. Exogenous UHMK1 was immunoprecipitated with the anti-HA antibody. Anti-HA, anti-FLAG and anti-GAPDH antibodies were used for western blot analyses. **(B)** CCK-8 assay in RKO cells transfected with VEC, STAT3-wt, STAT3-mut or UHMK1 overexpression plasmid in a combined way as indicated. *p<0.05, **P<0.01.

**Supplementary Table 1.** Primers used in this study for qRT-PCR and ChIP assay.
